# LARAI portal provides a safe method for lateral meniscus repair: three-dimensional computed tomography and cadaveric assessment

**DOI:** 10.1186/s10195-023-00727-1

**Published:** 2023-09-29

**Authors:** Yi Long, Zhengzheng Zhang, Min Zhou, Jingyi Hou, Yunfeng Zhou, Liang Jiang, Xiaoding Xu, Rui Yang

**Affiliations:** 1 Department of Orthopedics, Sun Yat-sen Memorial Hospital, Sun Yat-sen University, 107 Yan Jiang Road West, Guangzhou, 510120 Guangdong China; 2 Guangdong Provincial Key Laboratory of Malignant Tumor Epigenetics and Gene Regulation, Sun Yat-sen Memorial Hospital, Sun Yat-sen University, 107 Yan Jiang Road West, Guangzhou, 510120 Guangdong China

**Keywords:** LARAI portal, Meniscus, Popliteal artery, Posterior cruciate ligament, All-inside

## Abstract

**Background:**

Lateral, All-Round and All-Inside (LARAI) portal is a viewing or working portal for observing and repairing the lesions of the lateral meniscus. However, there are safety concerns about popliteal artery (PA) injuries during the procedure. This study aimed to assess the safe distance between the trajectory of the LARAI portal and PA.

**Materials and methods:**

Both three-dimensional computed tomography (3D-CT) and cadavers were used to simulate the LARAI portal trajectory. In the 3D-CT study, between January 2020 and September 2020, 45 participants who underwent computed tomography angiography were included in the study. The shortest distance from the PA to the simulated trajectory needle (PS) was measured using 3D-CT. Mean −3SD −2 was calculated to assess the safety of the LARAI portal trajectory. If this value was more than zero, the trajectory was considered “safe.” In the cadaveric study, lower limbs from seven fresh-frozen cadavers were used to establish the “safe” trajectories of the LARAI portal, and the PS was measured.

**Results:**

In the 3D-CT study, the longest PS (*P* < 0.001) was found 20 mm lateral to the edge of the patellar tendon trajectory at 0 mm from the posterior cruciate ligament (PCL). Safe trajectories were also found 10 mm, 15 mm, and 20 mm lateral to the edge of the patellar tendon at 0 mm from the PCL, as well as the 20 mm lateral to the edge of the patellar tendon at 3 mm from the PCL. The cadaveric study showed that the average PS of all safe trajectories closely adjoined to PCL was greater than 14 mm.

**Conclusions:**

The LARAI portal trajectory in the “figure of four” is safe, and the optimal insertion point is 10–20 mm lateral to the edge of the patellar tendon and closely adjoined to the posterolateral margin of the PCL at knee joint line level.

**Level of evidence:**

Level IV.

## Introduction

**Fig. 1:**
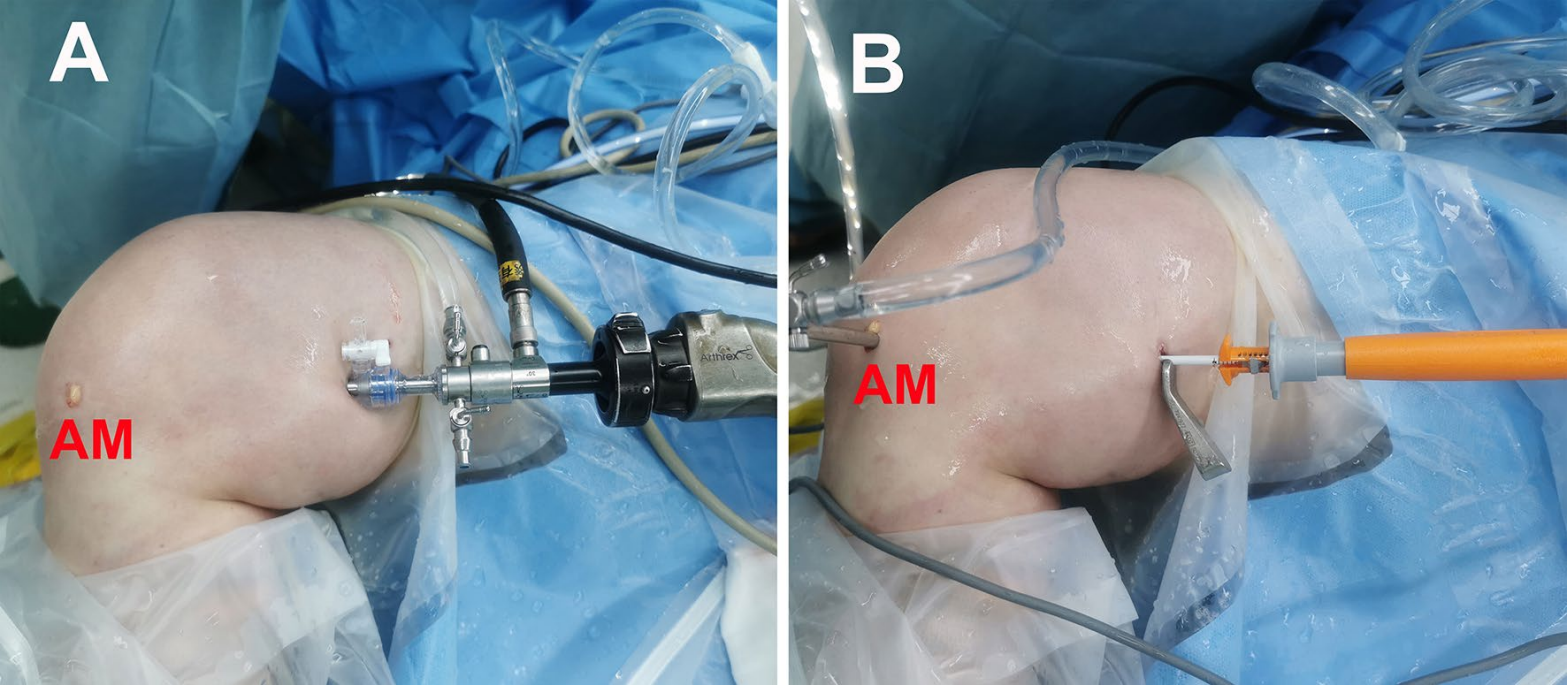
LARAI portal can serve as a working or viewing portal (right knee). **A** A viewing portal with the arthroscope through it. **B** A working portal with the FasT-Fix 360 system (Smith & Nephew, Inc. Andover, USA) through it. *AM* anteromedial portal

**Fig. 2:**
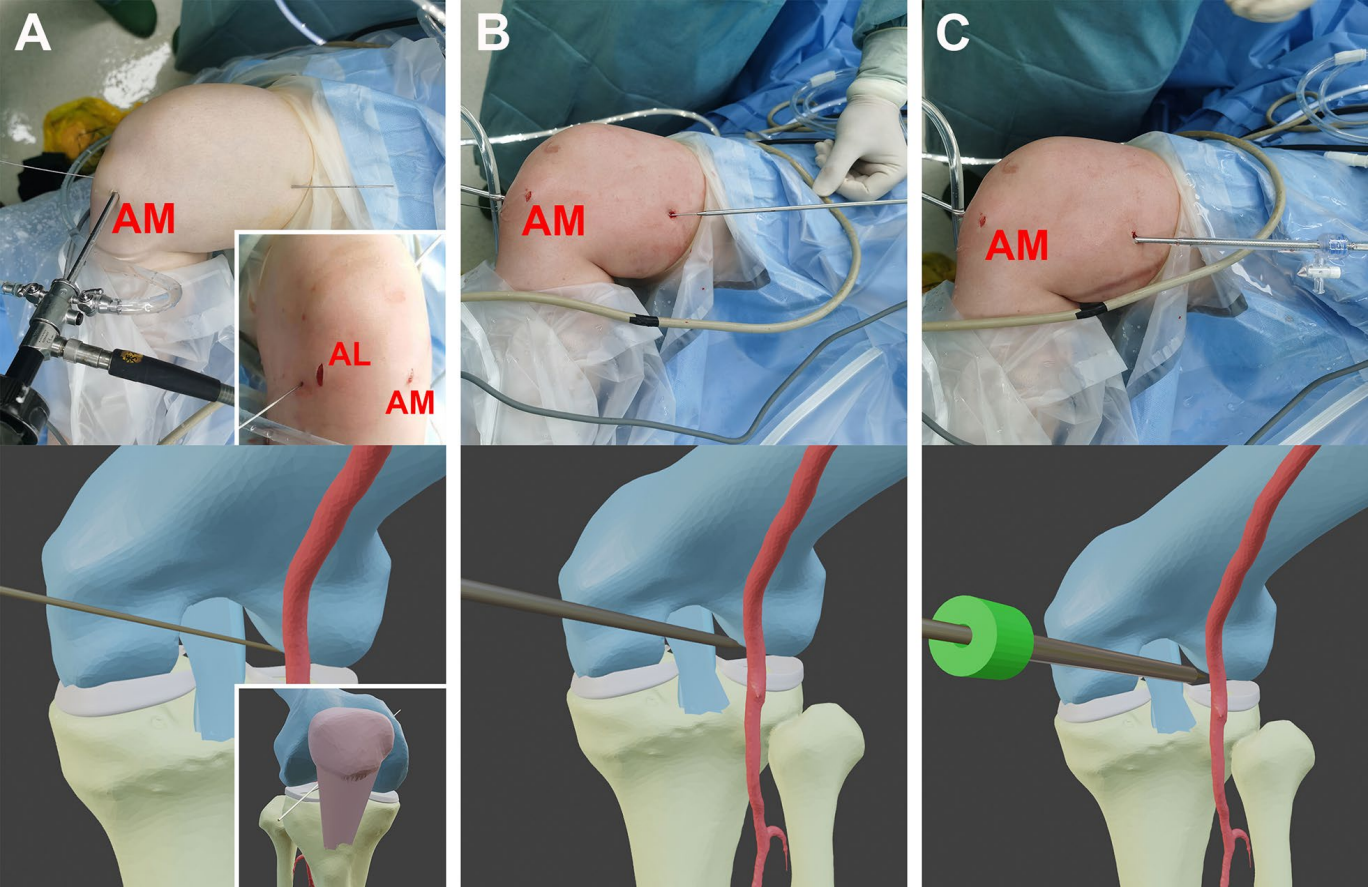
Establishment of the LARAI portal in the right knee. **A** A 1.5 mm diameter guide needle was inserted at a point 20 mm lateral to the patellar tendon, closely adjoining the posterolateral margin of posterior cruciate ligament, and pierced through the medial skin of distal thigh. **B** The trajectory was widened from the medial skin of distal thigh using a 3.5 mm diameter cannulated switching stick. **C** A 5.0 mm diameter slotted cannula (Hakko Corp, Nagano, Japan) was inserted into the articular cavity along the switching stick. *AL* anterolateral portal, *AM* anteromedial portal

The Lateral, All-Round and All-Inside (LARAI) portal is a viewing or working portal for observing and treating the lesions in the lateral meniscus (Fig. 1). It was first described in the Book of Endoscopy of the Hip and Knee [1]. The LARAI portal can be established by placing the patient in the “figure of four” position with the knee flexed at a 90° angle. Initially, a 1.5 mm diameter guide needle is inserted at a point 10–20 mm lateral to the patellar tendon at the joint line level, closely adjoined to the posterolateral margin of the posterior cruciate ligament (PCL) under arthroscopic visualization, and penetrating the medial skin of the distal thigh (Fig. 2A). Subsequently, the trajectory is expanded from the medial skin of the distal thigh along the inserted guide needle using a 3.5 mm diameter cannulated switching stick with a blunt end (Fig. 2B). Following this, a 5.0 mm diameter slotted cannula (Hakko Corp, Nagano, Japan) is introduced into the articular cavity along the switching stick (Fig. 2C). Finally, the switching stick and guide needle are removed, while the slotted cannula remains in place for the duration of the arthroscopic procedure.

**Fig. 3:**
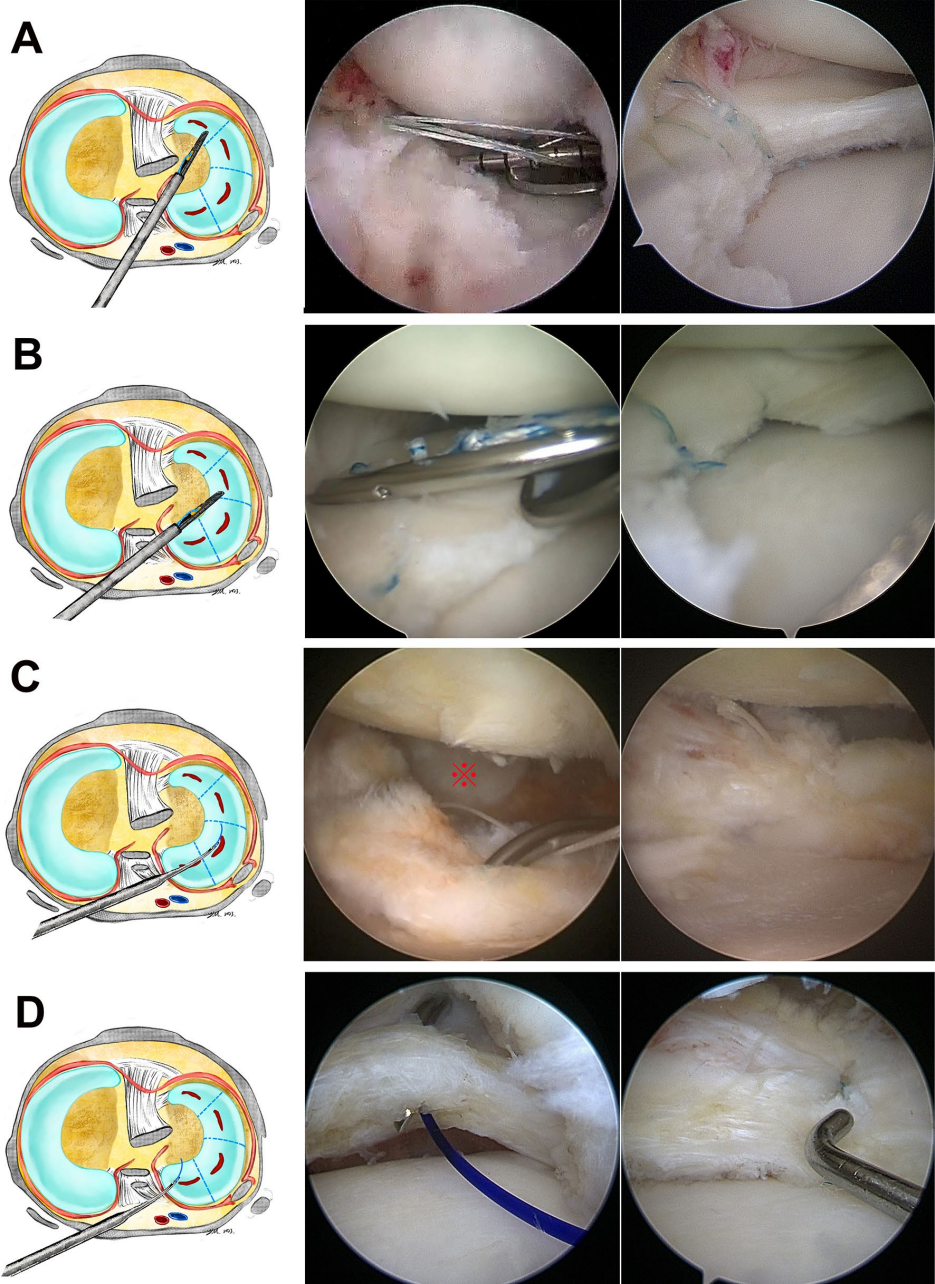
Schematic diagrams and perioperative images of the lateral meniscus repair in various segments (right knee). View from the anteromedial portal and all-inside instruments through the LARAI portal. **A** Anterior horn repair with FasT-Fix 360 system (Smith & Nephew, Inc. Andover, USA). **B** Middle segment repair with FasT-Fix 360 system. **C** Popliteal hiatus zone repair with a suture hook (Arthrex, Inc. Naples, USA). **D** Posterior horn repair with a suture hook. ref., popliteus tendonPlease confirm change.We have changed the sentence.

The trajectory of the LARAI portal passes from the medial skin of the distal thigh, through the posteromedial side of the knee joint, and adjoins the posterolateral border of the PCL, providing access to the lateral compartment of the knee joint. The authors named it LARAI portal due to the following reasons: (1) Lateral: this portal has been developed for the lateral meniscus; (2) All-Round: this portal can be used as a working or viewing portal for observing and treating various tears of any parts of the lateral meniscus; (3) All-Inside: this portal allows all-inside instrument, such as FasT-Fix 360 system (Smith & Nephew, Inc. Andover, USA) or suture hook (Arthrex, Inc. Naples, USA), to pass through, providing a broader application for all-inside techniques (Fig. 3). The greatest advantages of LARAI portal are: (1) providing enough view of the deep-seated inferior leaf of the anterior segment of the meniscus, especially in a discoid meniscus; (2) allowing all-inside devices to pass through for treating the tears extending to the anterior half of the lateral meniscus (AHLM) (Fig. 3A, B). Furthermore, this portal enables the suture hook to vertically suture the meniscus in the popliteal hiatus (PH) zone without capturing the capsule or anchoring to the popliteus tendon (Fig. 3C).

However, there are safety concerns about popliteal artery (PA) injuries when the guide needle inserts laterally to the edge of the patellar tendon and pierces through the medial skin of the distal thigh. In this study, we aimed to assess the safe distance between the LARAI portal trajectory and PA using both three-dimensional computed tomography (3D-CT) and cadavers. By employing these complementary methods, we aimed to enhance the reliability of our research outcomes and corroborate our findings. Some studies have reported that the PA is located posterolateral to the insertion point of the PCL [2–5]. They have also mentioned that PA injury is less likely when the knee is flexed to 90° since the PA is farther translocated posteriorly and laterally [6–9]. Consequently, we hypothesized that there is a safe distance between the trajectory of the LARAI portal and the PA in the “figure of four” position.

## Material and methods

This study was performed following the principles of the Declaration of Helsinki and approved by the Institutional Ethics Committee before initiation. Between January 2020 and September 2020, 45 patients diagnosed with lateral meniscus tears (21 male patients and 24 female patients with an average age of 35.0 years and an age range of 18–68 years) were included in this study. None of the patients had any of the following conditions: (1) previous surgery of the involved knee, (2) previous fracture of the distal femur or proximal tibia or knee dislocation, (3) diagnosis of PCL rupture, (4) concomitant congenital deformity of the knee, arteriovenous malformations, large popliteal cysts (d ≥ 5 cm), or other space-occupying lesions of the knee. All 45 patients included in this study underwent surgery, and some of them were treated using the LARAI portal technique. Lower limbs from seven fresh-frozen cadavers (4 males and 3 females with an average age of 69.3 years and an age range of 44–87 years) were used to establish the “safe” trajectories of the LARAI portal. None of them had previously undergone knee surgery or exhibited any knee deformities. (Table 1).

**Table 1:** Demographic characteristics of subjects

	Patient	Cadaver
Number	45	7
Age, mean ± SD (range)	35.02 ± 14.72 (18–68)	69.29 ± 14.93 (44–87)
Sex		
Female	24	3
Male	21	4
Laterality		
Right	23	2
Left	22	5

*SD* standard deviation

### 3D-CT study

**Fig. 4:**
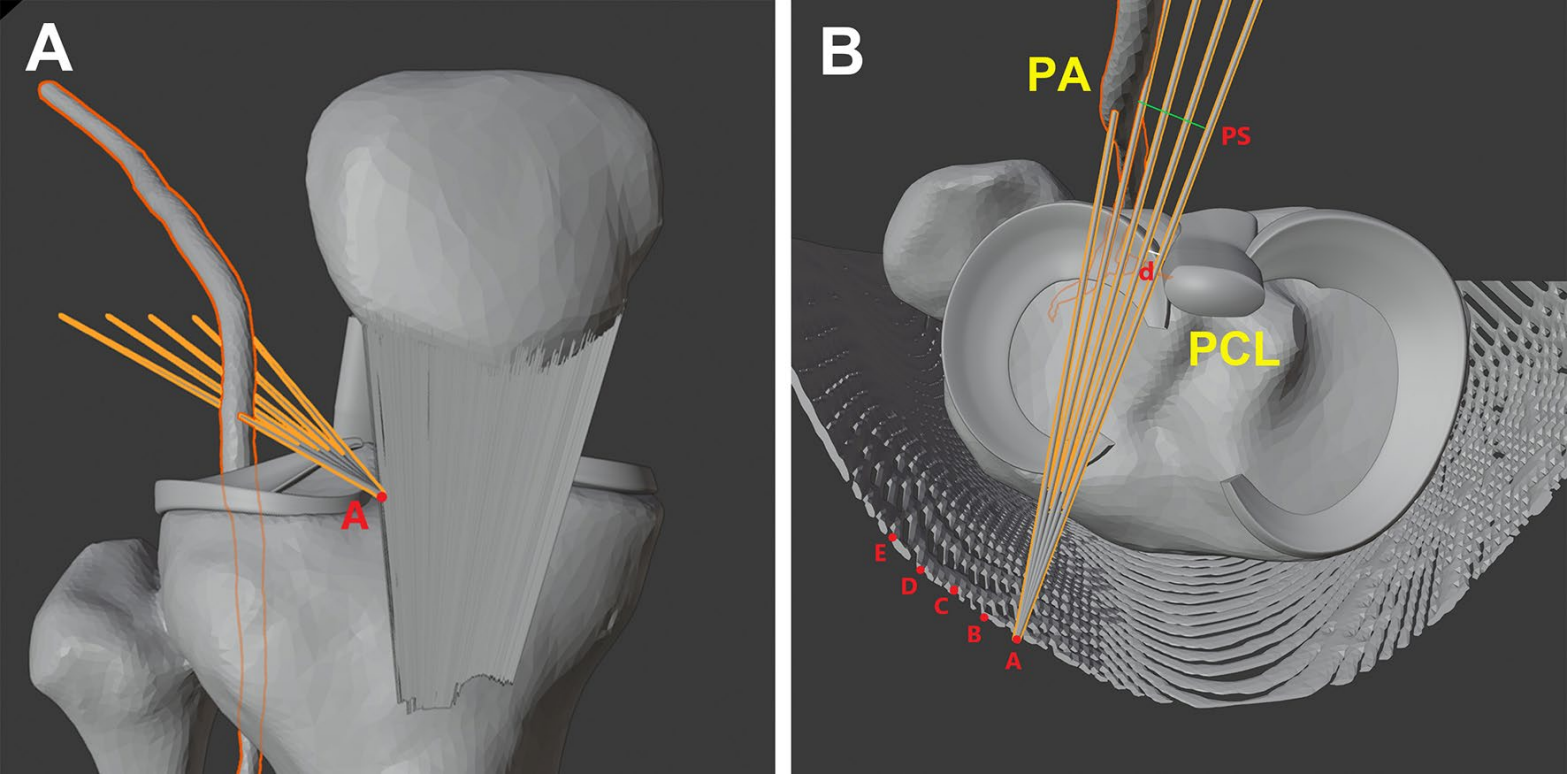
Measurements were performed using three-dimensional computed tomography (right knee). **A** Anterior view, **B** Axial view. *PA* popliteal artery, *PCL* posterior cruciate ligament, *PS* the shortest distance from the popliteal artery to the simulated trajectory needle, *d* the distance from the posterior cruciate ligament, *A–E* the insertion points 0 mm, 5 mm, 10 mm, 15 mm, or 20 mm lateral to the edge of the patellar tendon, respectively

In total, 45 patients underwent computed tomography angiography (CTA). Iohexol (Shanghai General Pharmaceutical Co., Ltd, Shanghai, China) was intravenously administered at a dose of 100 mL with a dual-syringe power injector. A dual-source CT scan device (Siemens Healthcare, Erlangen, Germany) was used and the knee was in the “figure of four” position. All data about the knee were obtained from Digital Imaging and Communications in Medicine (DICOM). 3D model was reconstructed using Mimics (Materialise, Leuven, Belgium). Then, 3D models were imported into Blender 2.81 (Amsterdam, the Netherlands) for analysis. The measurements were performed at joint line level. The simulated trajectory needles were inserted 0 mm, 5 mm, 10 mm, 15 mm, and 20 mm lateral to the edge of the patellar tendon (marked with A, B, C, D, E). For each insertion point, there were five trajectory needles with increasing distances from the PCL (0 mm, 3 mm, 6 mm, 9 mm, and 12 mm). The shortest distance from the PA to each simulated trajectory needle (PS) was measured in millimeters (Fig. 4).

### Cadaveric study

**Fig. 5:**
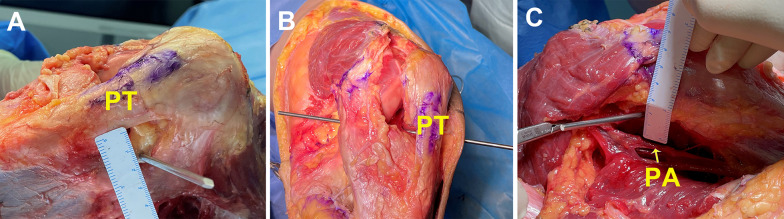
“Safe” trajectories of the LARAI portal in a fresh-frozen cadaveric knee at 90° flexion (left knee). **A** The puncture needle was inserted 15 mm lateral to the edge of the patellar tendon. **B** The puncture needle was closely adjoined to the posterolateral margin of the posterior cruciate ligament under direct visualization. **C** The distance from the puncture needle to the PA. *PA* popliteal artery, *PT* patellar tendon

To confirm the results, lower limbs from seven fresh-frozen cadavers were used to establish the “safe” trajectories. The knee was 90° flexed during the surgical procedure. The skin around the knee was removed to expose the patellar tendon. Two longitudinal incisions, medial and lateral to the patellar tendon, were made to open the knee joint. The hamstring tendon in the posteromedial position was cut to identify the PA. The puncture needle was inserted 10 mm, 15 mm, or 20 mm lateral to the edge of the patellar tendon and closely adjoined to the posterolateral margin of the PCL under direct visualization. The shortest distance from the PA to the puncture needle was measured in millimeters (Fig. 5).

### Statistical analysis

All data from the 3D model study were measured by two independent radiologists and repeated by another researcher after 3 weeks. Interclass correlation coefficients (ICC) were assessed by examining inter- and intraobserver agreement. The one-way repeated measures analysis of variance was used to compare the distance from the PA to the trajectory at different insertion points. In addition, the distance from each insertion point to other insertion points at each distance from the PCL was also measured. The value mean −3SD −2 was used for assessing the safety of trajectories. The point mean −3SD statistically includes 99.87% of all values for a single tail normal distribution. In addition, 2 mm was added to mean −3SD as the “safe distance” [10]. If the value of mean −3SD −2 was more than zero, 99.87% of patients were considered at no risk of PA injury during the LARAI portal; therefore, the simulated trajectory was considered “safe.” *P* < 0.05 was considered statistically significant. The variables are presented as mean and standard deviation (mean ± SD). All statistical analyses were performed using SPSS (Version 19, IBM Corp, USA).

## Results

### 3D model measurement

The interobserver ICC for the 3D model measurement was 0.989 (95% CI 0.988–0.990), and the intraobserver ICC was 0.994 (95% CI 0.993–0.995).

**Fig. 6:**
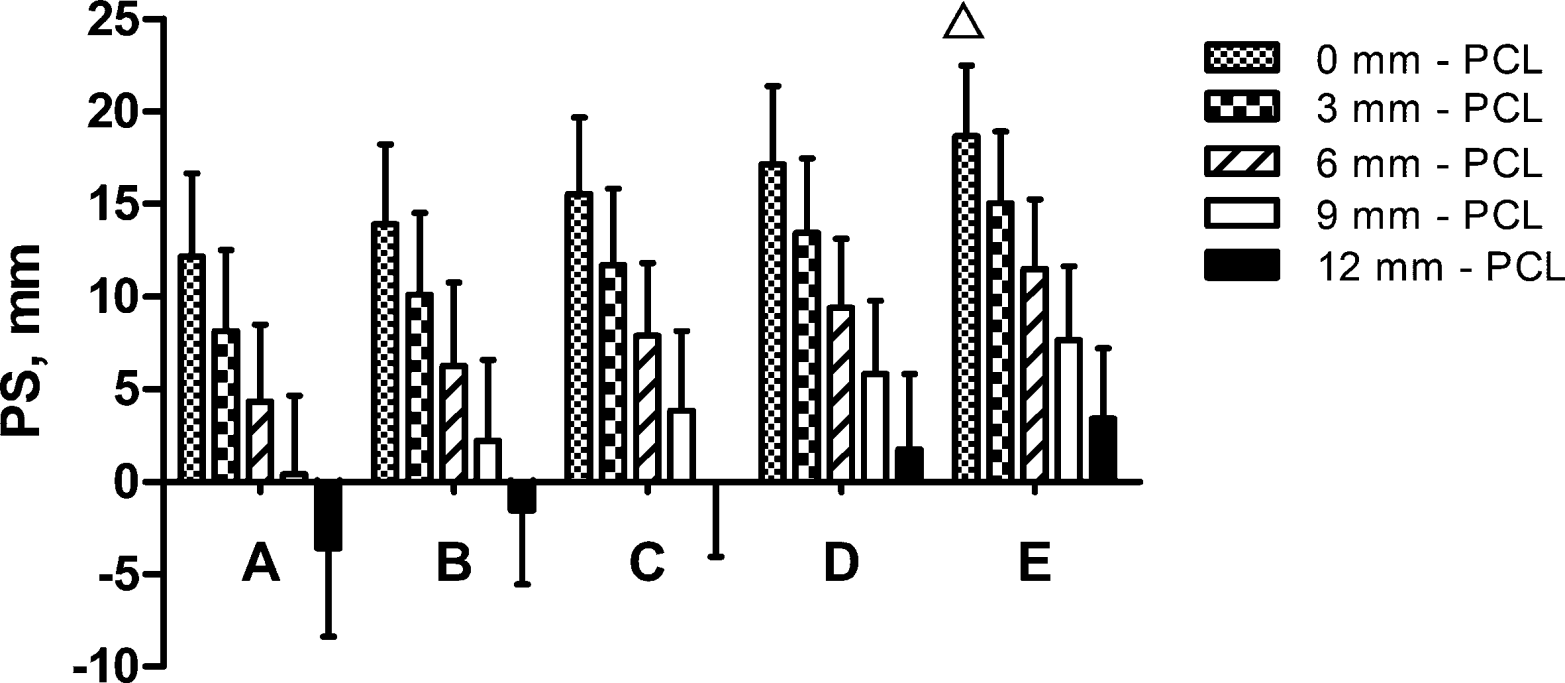
Comparing the distance from the PA to the simulated trajectory needles. The trajectory initiating 20 mm lateral to the edge of the patellar tendon at 0 mm from the PCL had the longest PS (△). The bars show the mean and standard deviation. A–E, the insertion points 0 mm, 5 mm, 10 mm, 15 mm, or 20 mm lateral to the edge of the patellar tendon, respectively. *PA* popliteal artery, *PCL* posterior cruciate ligament, *PS* the shortest distance from the popliteal artery to the simulated trajectory needle

There were five insertion points (A–E) lateral to the edge of the patellar tendon. Five simulated trajectory needles with increasing distances from the PCL were inserted through each point. Compared with other insertion points at each distance from the PCL, insertion point E (20 mm lateral to the edge of the patellar tendon) had the longest distance of PS (*P* < 0.001). Compared with other insertion points at each distance from the PCL, the trajectory closely adjoined to PCL (0 mm from the PCL) had the longest distance of PS (*P* < 0.001). Therefore, the trajectory initiating from 20 mm lateral to the edge of the patellar tendon with 0 mm distance from the PCL had the longest distance of PS (Fig. 6).

The trajectories initiating from 10 mm, 15 mm, and 20 mm lateral to the edge of the patellar tendon with 0 mm distance from the PCL were considered safe, and their average PS was 15.53 ± 4.15 mm, 17.14 ± 4.22 mm, and 18.67 ± 3.80 mm. The trajectory initiating 20 mm lateral to the edge of the patellar tendon with 3 mm distance from the PCL was considered a safe trajectory with an average PS of 15.05 ± 3.88 mm. All data are summarized in Table 2.

**Table 2:** Distances between the PA and the simulated trajectory needles

Distance of simulated trajectory to PCL	0 mm lateral to PT	5 mm lateral to PT	10 mm lateral to PT	15 mm lateral to PT	20 mm lateral to PT
0 mm					
Mean ± SD	12.14 ± 4.51	13.90 ± 4.32	15.53 ± 4.15	17.14 ± 4.22	18.67 ± 3.80
Range	4.32–22.97	5.86–25.09	7.64–25.11	9.23–28.18	10.49–26.94
Mean −3SD −2	−3.39	−1.09	1.08	2.48	5.27
Safe	No	No	**Yes**	**Yes**	**Yes**
3 mm					
Mean ± SD	8.13 ± 4.38	10.07 ± 4.44	11.68 ± 4.15	13.43 ± 4.03	15.05 ± 3.88
Range	0.98–18.59	2.06–19.50	3.25–23.10	5.95–25.00	7.83–25.55
Mean −3SD −2	− 7.01	− 5.25	− 2.77	− 0.66	1.41
Safe	No	No	No	No	**Yes**
6 mm					
Mean ± SD	4.33 ± 4.16	6.26 ± 4.48	7.86 ± 3.93	9.37 ± 3.73	11.47 ± 3.78
Range	−3.03 to 13.86	−2.15 to 16.46	0.9–16.89	2.59–18.19	4.32–19.52
Mean −3SD −2	−10.15	−9.18	−5.93	−3.82	−1.87
Safe	No	No	No	No	No
9 mm					
Mean ± SD	0.39 ± 4.28	2.20 ± 4.38	3.84 ± 4.29	5.81 ± 3.94	7.62 ± 4.01
Range	−6.44 to 11.54	−5.42 to 12.48	−3.99 to 15.13	−0.54 to 16.74	−0.82 to 16.39
Mean −3SD −2	−14.45	−12.94	−11.03	−8.01	−6.41
Safe	No	No	No	No	No
12 mm					
Mean ± SD	−3.60 ± 4.78	−1.55 ± 4.01	−0.02 ± 4.04	1.73 ± 4.08	3.39 ± 3.79
Range	−14.06 to 8.43	−8.85 to 9.55	−8.98 to 10.41	−5.19 to 11.12	−4.21 to 11.67
Mean −3SD −2	−19.94	−15.58	−14.14	−12.51	−9.98
Safe	No	No	No	No	No

All measurements are in millimeters. If the value of Mean −3SD −2 was more than zero, the simulated trajectory was considered “safe”

*PA* popliteal artery, *PCL* posterior cruciate ligament, *PT* patellar tendon, *SD* standard deviation

### Cadaveric measurement for “safe” trajectories

The trajectories initiating 10 mm, 15 mm, and 20 mm lateral to the edge of the patellar tendon with 0 mm distance from the PCL (closely adjoined to PCL) were established in the lower limbs of seven fresh-frozen cadavers. The average PS of trajectories initiating from 10 mm, 15 mm, or 20 mm lateral to the edge of the patellar tendon was 14.00 ± 3.27 mm, 16.43 ± 2.94 mm, and 18.29 ± 2.81 mm, respectively. (Table 3).

**Table 3:** Cadaveric measured distances between the PA and the “safe” trajectories of the LARAI portal

Specimen	10 mm lateral to PT	15 mm lateral to PT	20 mm lateral to PT
1	13	15	16
2	17	19	20
3	14	16	17
4	12	15	17
5	19	21	23
6	9	12	15
7	14	17	20
Mean ± SD	14.00 ± 3.27	16.43 ± 2.94	18.29 ± 2.81

All measurements are in millimeters

*PA* popliteal artery, *PT* patellar tendon, *SD* standard deviation

## Discussion

Importantly, this study found that there is a safe distance between the trajectory of the LARAI portal and PA.

Gupta et al. reported that the distance of 2 mm from the popliteal neurovascular bundle (PNVB) is empirically a safe distance. They statistically explained that a mean −3SD −2 value of more than zero is safe in 99.87% of cases [10]. We used their method to assess vascular safety during the establishment of the LARAI portal. Our 3D-CT results showed that more than 99.8% of cases were at no risk of PA injury when the puncture needle was inserted 10–20 mm lateral to the edge of the patellar tendon at 0 mm from the PCL. The cadaveric study showed that all specimens had a safe distance between the PA and the puncture needle. Our final analysis demonstrates consistency between the results obtained from both complementary methods. Compared with Gupta’s study [10], we only assessed the safety of PA. Some studies reported that the PA is the most anterior and medial structure in the PNVB [2, 11]. Therefore, the safety of the PA during the establishment of the LARAI portal theoretically ensures the safety of the whole PNVB.

Herein, we used 3D-CT instead of magnetic resonance imaging (MRI) to determine the safe distance. Compared with MRI, 3D-CT may be more accurate for measuring the safe distance because the Blender software can provide more visualized and real 3D images to simulate the LARAI portal for analyzing the risk of PA injury.

**Fig. 7:**
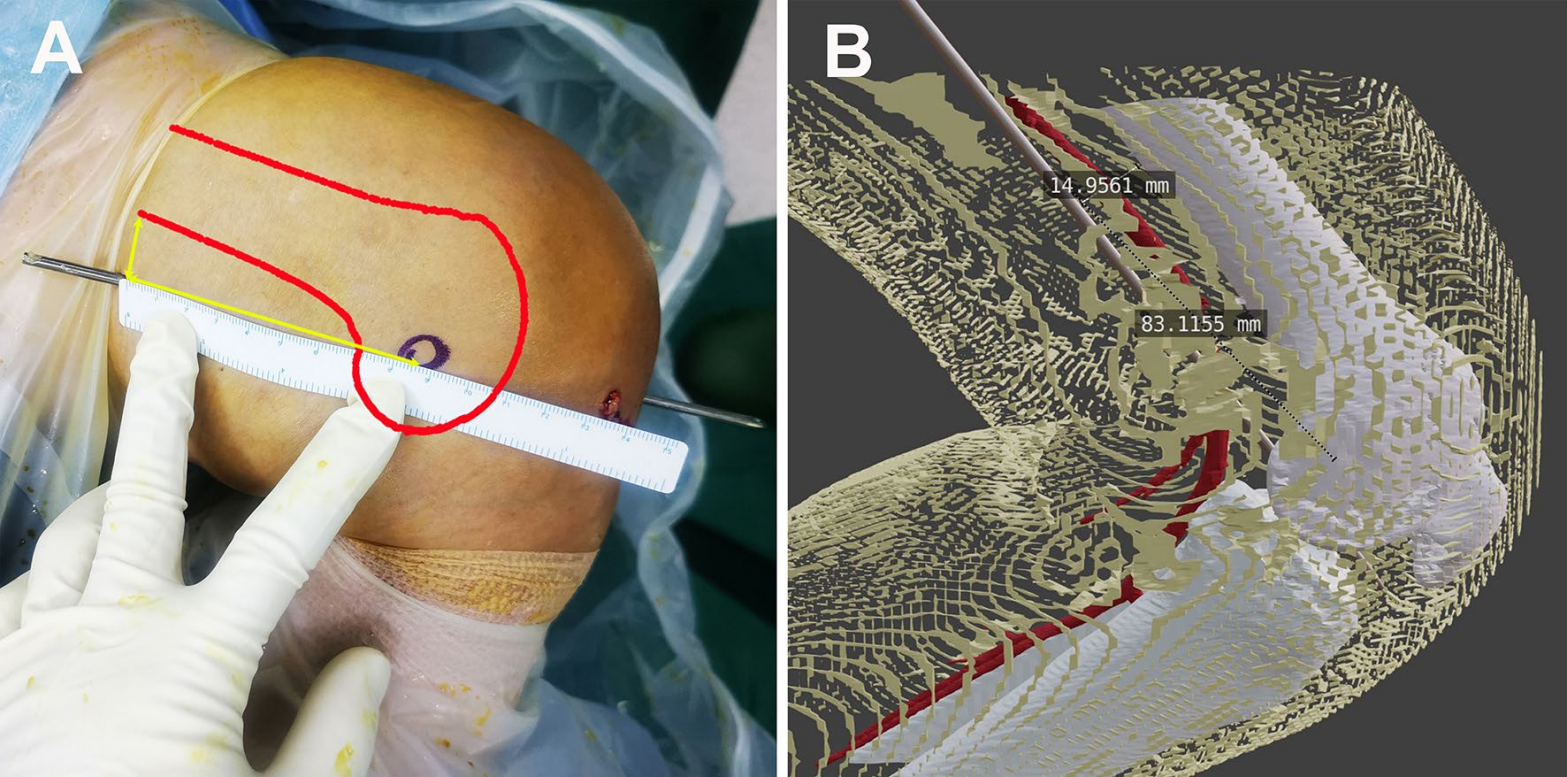
Clinical illustration shows the position of the exit point of the needle (left knee). Distances between the exit point and medial epicondyle of the femur is approximately 85 mm, and distances between the exit point and the surface projection of the posterior femoral margin is approximately 15 mm. **A** Measurement in a patient. **B** Measurement in three-dimensional computed tomography

In the present study, we also found that 20 mm lateral to the edge of the patellar tendon at 0 mm from the PCL was the safest trajectory. The trajectory might remain safe at longer distances, for example, 25 mm or more lateral to the edge of the patellar tendon. However, the trajectories with great distances from the edge of the patellar tendon may damage the lateral condyle of the femur. Thus, we recommend 10–20 mm lateral to the edge of the patellar tendon at the level of the knee joint line as the optimal insertion point for the LARAI portal. Consequently, the exit point of the needle would be the medial skin of the distal thigh, approximately 70–90 mm proximal to the medial epicondyle of the femur and 15 mm behind the surface projection of the posterior femoral margin (Fig. 7).

The resection of the inferior leaf of the anterior half of the lateral meniscus (AHLM) through routine arthroscopic portals is challenging for arthroscopic surgeons. Particularly, tears involving the anterior horn of the lateral discoid meniscus are the main problem [12–14]. Several accessory portals, including inframeniscal, lateral patellofemoral auxiliary, high anteromedial, or far anteromedial portal, have been proposed to improve the visualization and repair of AHLM [15–19]. However, these were still insufficient to observe the deep-seated inferior leaf of the anterior horn meniscus and potentially increased the risk of meniscus injury. In addition, due to their improper insertion angle, surgeons rarely used these accessory portals to perform all-inside device repairs of tears extending to the AHLM. To date, the outside-in method remains the standard strategy for repairing the anterior horn of the meniscus [20]. However, it requires a skin incision to bury the suture thread, and if the stitch is made over the lateral fascia with a non-absorbable monofilament wire, it may cause knots protrusion or local pain due to tissue strangulation and knots irritation [21–24]. The LARAI portal is an alternative method for repairing AHLM by all-inside devices under direct arthroscopic visualization (Fig. 3A, B).

Using an all-inside device for repairing lateral meniscus tears is still controversial. Ouanezar et al. found that the popliteus tendon provides a safe and reliable location for all-inside meniscal repair device placement with a low failure rate and no complications [25]. However, some authors have reported that the thin capsule around the PH makes the sutures less efficient. Additionally, sutures of the popliteus tendon with an all-inside device may become loose or cause pain and irritation during knee motion [14, 26–28]. Furthermore, Chuaychoosakoon et al. demonstrated that repairing the lateral meniscus using the all-inside meniscal device through either the anterolateral portal or the anteromedial portal can endanger the peroneal nerve or popliteal artery [29]. The LARAI portal helps to vertically stitch the meniscus in the PH zone and posterior horn without capturing the popliteus tendon or capsule (Fig. 3C, D). Therefore, it can theoretically prevent over-constraining the meniscus and surrounding normal tissues, and avoid iatrogenic neurovascular injury (including peroneal nerve) caused by the tip of the all-inside device.

There were several limitations to this study. First, the presence of anatomic variances, popliteal lumps, such as Baker’s cysts and posterior osteophytes, may alter the anatomy of the PA; therefore, careful preoperative evaluation of PA by MRI is necessary. Second, patients’ characteristics such as age, sex, body mass index (BMI), and the side of the knee should be taken into account. Some studies indicated that the anatomy of the PA does not significantly change by height, weight, BMI, sex, or side of the knee [2, 5]. However, Gilat et al. reported that young and female patients may be at greater risk of PNVB injury [30]. Lastly, we did not assess the anatomical relationship between the trajectory and the saphenous nerve (SN). Sharp knives should be only used to reduce the risk of SN injury during the establishment of the LARAI portal. The trajectory can then be broadened by a blunt switching stick or slotted cannula.

In conclusion, the establishment of the LARAI portal in the “figure of four” position is safe. The optimal insertion point is 10–20 mm lateral to the edge of the patellar tendon and closely adjoined to the posterolateral margin of the PCL at knee joint line level.

## Data Availability

The datasets used during the current study are available from the corresponding author on reasonable request.

## Abbreviations

PA: Popliteal artery
PS: Shortest distance from PA to simulated trajectory needle
3D-CT: Three-dimensional computed tomography
PCL: Posterior cruciate ligament
AHLM: Anterior half of the lateral meniscus
PH: Popliteal hiatus
CTA: Computed tomography angiography
DICOM: Digital imaging and communications in medicine
ICC: Interclass correlation coefficients
PNVB: Popliteal neurovascular bundle
MRI: Magnetic resonance imaging
SN: Saphenous nerve
